# *BCAT1*: A risk factor in multiple cancers based on a pan‐cancer analysis

**DOI:** 10.1002/cam4.4525

**Published:** 2022-01-04

**Authors:** Guo‐Sheng Li, He‐Qing Huang, Yao Liang, Qiu‐Yu Pang, Hao‐Jia Sun, Zhi‐Guang Huang, Yi‐Wu Dang, Lin‐Jie Yang, Gang Chen

**Affiliations:** ^1^ Department of Pathology The First Affiliated Hospital of Guangxi Medical University Nanning China

**Keywords:** cancer biology, cancer risk factors, immunology, prognosis, target therapy

## Abstract

**Background:**

Although branched chain amino acid transaminase 1 (*BCAT1*) has been identified to play an essential role in multiple tumors, no studies on its role in pan‐cancer have been consulted before.

**Methods:**

The study comprehensively analyzes the expression, potential mechanisms, and clinical significance of *BCAT1* in pan‐cancer through utilizing 16,847 samples, providing novel clues for the treatment of cancers. A Kruskal–Wallis test and the Wilcoxon rank‐sum and signed‐rank tests were applied to investigate diverse *BCAT1* expression between various groups (e.g., cancer tissues versus normal tissues). Spearman’s rank correlation coefficient was used in all correlation analyses in the study. Cox analyses and Kaplan‐Meier curves were utilized to identify the prognosis significance of *BCAT1* expression in cancers. The significance of *BCAT1* expression in differentiating cancer and non‐cancer tissues was explored via the area under the receiver operating characteristic curves (AUC).

**Results:**

The differential expression of *BCAT1* was detected in various cancers (*p* < 0.05), which is relevant to some DNA methyltransferases expression. *BCAT1* expression was associated with mismatch repair gene expression, immune checkpoint inhibitors expression, microsatellite instability, and tumor mutational burden in some cancers, indicating its potential in immunotherapy. *BCAT1* expression showed prognosis significance and played a risk role in multiple cancers (hazard ratio > 0, *p* < 0.05). *BCAT1* expression also demonstrated conspicuous ability to distinguish some cancers tissues from their normal tissues (AUC > 0.7), indicating its potential to detect cancers. Further analyses on head and neck squamous cell carcinoma certified upregulated BCAT1 expression at both mRNA and protein levels in this disease based on in‐house tissue microarrays and multicenter datasets.

**Conclusions:**

For the first time, the research comprehensively demonstrates the overexpression of *BCAT1* in pan‐cancer, which improves the understanding of the pathogenesis of *BCAT1* in pan‐cancer. Upregulated *BCAT1* expression represented a poor prognosis for cancers patients, and it serves as a potential marker for cancer immunotherapy.

## Graphical abstract

For the first time, the research comprehensively demonstrates over‐expression of BCAT1 in pan‐cancer, which improves the understanding of the pathogenesis of BCAT1 in pan‐cancer. Upregulated BCAT1 expression represents poor prognosis of cancers patients, and it serves as a potential marker for cancer immunotherapy.

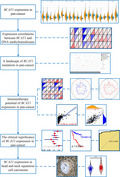

## 1 INTRODUCTION

Cancer is an increasingly significant and serious burden to human health, representing one of the most common causes of death. According to the predicted data for China, more than 4.6 million persons were diagnosed with cancer and more than 2.9 million patients would die in 2018.^1^ Globally, there were estimated 19.3 million newly diagnosed cases and nearly 10.0 million deaths from various cancers in 2020.^2^ With clinical care for cancers, such as multidisciplinary methods, including surgery, radiotherapy, chemotherapy, and targeted therapy, progression in treatment effects for some cancers is significant, such as melanoma and Hodgkin lymphoma.^3^ However, the mortality rates in some cancers remain dismal, such as in pancreatic cancer, which has an estimated mortality rate almost equal to its predicted diagnosed cases in the same year.^2^ In terms of the pathogenesis of cancers, the known risk factors include tobacco consumption, alcohol consumption, and obesity.^4^, ^5^ However, the pathogenesis of cancer is very complex and far from fully elucidated. Thus, it is necessary to explore the mechanism of cancer occurrence and progression to identify potential cancer‐related biomarkers for the treatment of cancer.

Branched chain amino acid transaminase 1 (*BCAT1*) is located at human chromosome 12p12.1. According to previous studies, it plays an important role in the development of tumors. *BCAT1* has been identified as being highly expressed in a variety of cancers, such as gliomas, gastric cancer, and hepatocellular carcinoma, and has been frequently reported as a promoter in the occurrence and development of these cancers.^6^, ^7^, ^8^, ^9^, ^10^ Moreover, in glioma,^11^ prostate cancer,^12^ and urothelial cancer,^13^ it has been demonstrated that the prognosis of tumor patients with upregulated *BCAT1* expression was poor. Nevertheless, the comprehensive potential mechanism and clinical significance of *BCAT1* in pan‐cancer have not previously been reported, and more efforts are needed to address this lack.

In this study, for the first time, the expression, potential mechanism, and clinical significance of *BCAT1* in pan‐cancer were explored based on data from multiple sources including the Genotype‐Tissue Expression (GTEx), Cancer Cell Line Encyclopedia (CCLE), The Cancer Genome Atlas (TCGA), and Gene Expression Omnibus (GEO) databases, as well as the published literature. Furthermore, we analyzed *BCAT1* expression in head and neck squamous cell carcinoma (HNSCC) based on larger samples from in‐house tissue microarrays and public datasets. Overall, the research highlights an essential role of *BCAT1* expression in pan‐cancer, providing a novel and potential therapeutic target for various cancers.

## 2 MATERIALS AND METHODS

### 2.1 *BCAT1* expression data in pan‐cancer

GTEx^14^ collects numerous normal tissues of *homo sapiens*, and this collection was applied to explore *BCAT1* expression in 30 kinds of normal tissues in the study. CCLE^15^ is a database feasible for accessing gene expression in multiple cell lines of cancers. For this purpose, CCLE data were collected for analyzing *BCAT1* expression in cancers included in the TCGA dataset. On November 16, 2021, GTEx data (*n* of samples = 8671) and CCLE data (*n* of samples = 473) were downloaded from the GTEx Portal and DepMap Portal, respectively.

TCGA contains large samples of tumors and their normal samples, and the data of 32 cancers from the database were included in this study. Twenty of the 32 cancers from TCGA were identified with both cancer tissues and normal tissues, and thus data of the 20 cancers were utilized to analyze *BCAT1* expression. The TCGA samples were obtained from the Xena database (constructed by the University of California, Santa Cruz) on November 16, 2021. Three types of samples (i.e., primary tumor, solid tissue normal, and primary blood‐derived cancer peripheral blood; *n* of cancers = 9054; *n* of controls = 727) from TCGA were included. The details of the 32 cancers are listed in Appendix S1.

### 2.2 DNA methyltransferases and *BCAT1* mutations data

The expression levels of three typical DNA methyltransferases (*DNMT1*, *DNMT3A*, and *DNMT3B*) were obtained from the TCGA data. A *BCAT1* mutations dataset, processed via MuTect2 software,^16^ came from GDC Portal. A landscape of *BCAT1* mutations in pan‐cancer was generated based on the dataset, and the process was completed using Sanger Box (v3.0).

### 2.3 Data of mismatch repair genes, immune checkpoint genes, microsatellite instability, tumor mutational burden, neoantigens count, and immune microenvironment

The expression levels of five mismatch repair (MMR) genes (*MLH1*, *MSH2*, *MSH6*, *PMS2*, and *EPCAM*) and 46 immune checkpoints (*BTLA*, etc.) were extracted from the TCGA data. Microsatellite instability (MSI), tumor mutational burden (TMB), and neoantigens count (containing single‐nucleotide variation and indel neoantigens)^17^ data were obtained from the previous study by Liu et al.^18^

The infiltration level data of six kinds of immune cells (B cells, CD4^+^ T cells, CD8^+^ T cells, neutrophils, macrophages, and dendritic cells) were downloaded from TIMER.^19^ Three scores (immune score, stromal score, and ESTIMATE score) of the immune microenvironment were calculated via the ESTIMATE^20^ algorithm.

### 2.4 Clinical correlation of *BCAT1* in pan‐cancer

The clinical information of cancer patients was obtained from the Xena database and included clinical features and prognosis data. The correlations of *BCAT1* expression with three clinical features—age, gender, and American Joint Committee on Cancer stage—were assessed using Wilcoxon tests.

Overall survival time (OST), disease‐specific survival time (DSST), disease‐free interval time (DFIT), and progression‐free interval time (PFIT) are common clinical indicators in evaluating patients’ prognoses. The criterion for censoring OST is death by any cause. DSST is identified when a patient dies of a specific disease. DFIT indicates a period without signs of cancer recurrence, while PFIT is a measure of time when patients live with diseases without worsening or advancement of the disease. Univariate Cox analysis was utilized to explore the relationship between *BCAT1* expression and OST. For one cancer, when the *p* value of univariate Cox analysis was < 0.05, the relevance between *BCAT1* expression and the OST of patients with this cancer was further analyzed through a Kaplan–Meier curve. Similar to OST, the correlations between *BCAT1* expression and three prognosis signatures (DSST, DFIT, and PFIT) were investigated via univariate Cox analysis and Kaplan–Meier curves.

The significance of *BCAT1* expression in differentiating cancer and non‐cancer tissues was explored via the area under the receiver operating characteristic curves (AUC). The greater the AUC value (ranging from 0 to 1), the more remarkable the ability of *BCAT1* expression to differentiate cancer tissues from non‐cancer tissues, which implies *BCAT1*’s potential as a marker for cancer screening.

### 2.5 Further analyses of *BCAT1* expression in HNSCC

To verify *BCAT1* mRNA expression in HNSCC, multicenter microarrays were collected from the GEO database. The screening strategy was as follows: “(larynx OR mouth OR pharynx OR (Head and Neck Neoplasms)) AND (mRNA OR gene).” The inclusion criteria for the datasets were as follows: (1) the species was *homo sapiens*; (2) samples were from HNSCC tissue or HNSCC cell line; (3) each dataset included HNSCC samples or non‐HNSCC control samples; and (4) each dataset included *BCAT1* expression profiling data. Exclusion criteria included the following: duplicate samples or incomplete data. A total of 44 microarray datasets were collected from GEO, containing HNSCC and 633 non‐HNSCC samples. After removing batch effects between diverse datasets using a surrogate variable analysis software package,^21^ the samples were divided into four subgroups––laryngeal squamous cell carcinoma (LSCC), oral squamous cell carcinoma (OSCC), pharyngeal squamous cell carcinoma (PSCC), and unspecific site head and neck squamous cell carcinoma (USCC). The information of samples from the GEO database is provided in Appendix S2.

To investigate BCAT1 protein levels in HNSCC, an immunohistochemistry (IHC) experiment was performed based on five in‐house tissue microarrays (TOC481, ORC1021, HNT961, HNT962, and HNT1021), which contained 258 samples and was provided by Pantomics, Inc (Richmond, CA 94806). Similar to mRNA data, BCAT1 protein data were also classified into four types—LSCC, OSCC, PSCC, and USCC (all belonging to HNSCC). The clinical characteristics of the samples are shown in Appendix S3.

All steps of the IHC experiment were conducted according to the instructions of the manufacturer. The dewaxed and repaired tissue slides were placed in 0.01 M citrate buffer solution (pH = 6.0) to extract the antigen. The endogenous peroxidase was inactivated with 3% H_2_O_2_. The tissue slides were incubated overnight in the rabbit anti‐human BCAT1 monoclonal antibody (ab126762, ABCAM, UK; dilution 1:100) at 4°C, whereas the negative control slides were incubated overnight in a phosphate buffer. A second antibody‐labeled horseradish peroxidase (Changdao Biotechnology Co., Ltd., Shanghai, China) was added to the tissue slides, and the latter were then stored at room temperature (approximately 25℃) for 25 min and colored with 3,3’‐diaminobenzidine. The dehydrated and sealed slides were evaluated under a brightfield microscope. Positive staining appeared as brown particles in the nucleus and/or cytoplasm, whereas negative staining appeared as blue particles. All samples of the tissue microarrays were randomly selected from 10 regions to evaluate the positive cells; this was performed independently by two authors. The standard of the staining intensity score was as follows: integers 0–3 scores, for no staining, light staining, moderate staining, and strong staining, respectively; for positive cells, integers 0–4 scores, for <5%, 5%–25%, 26%–50%, 51%–75%, and >75%, respectively, of the positive cells of the visual field. The total IHC score was calculated as the product of the intensity score and the positive cells score, and the final total IHC score was the average of the two evaluations. Through the experiment and total IHC scores of the clinical samples, we attempted to analyze the difference in BCAT1 protein levels between the HNSCC and non‐HNSCC groups.

### 2.6 Statistical analysis

A Kruskal–Wallis test was utilized to explore the difference in *BCAT1* expression between various normal human tissues from GTEx, and the same method was also used for cancer cell lines from CCLE. The Wilcoxon rank‐sum and signed‐rank tests were applied to investigate diverse *BCAT1* expression between cancers and their normal tissues. Spearman's rank correlation coefficient was used in correlation analyses of *BCAT1* expression with DNA methyltransferases expression, MMR genes expression, immune checkpoints expression, MSI, TMB, neoantigens, immune cell infiltration levels, and several immune‐related scores, and all correlation analyses were based on data of 32 cancers from TCGA.

The standardized mean difference (SMD) of the forest plot and Wilcoxon tests of violin plots were used to assess the difference in *BCAT1* expression levels between the HNSCC group and the non‐HNSCC group. In calculating SMD, a fixed‐effects model was used when an *I^2^* value of the *I^2^* test was ≤50%; otherwise, a random‐effects model was applied. An SMD value greater than zero suggested upregulated *BCAT1* expression in the HNSCC group, while a value less than zero represented low *BCAT1* expression in HNSCC.

**FIGURE 1:**
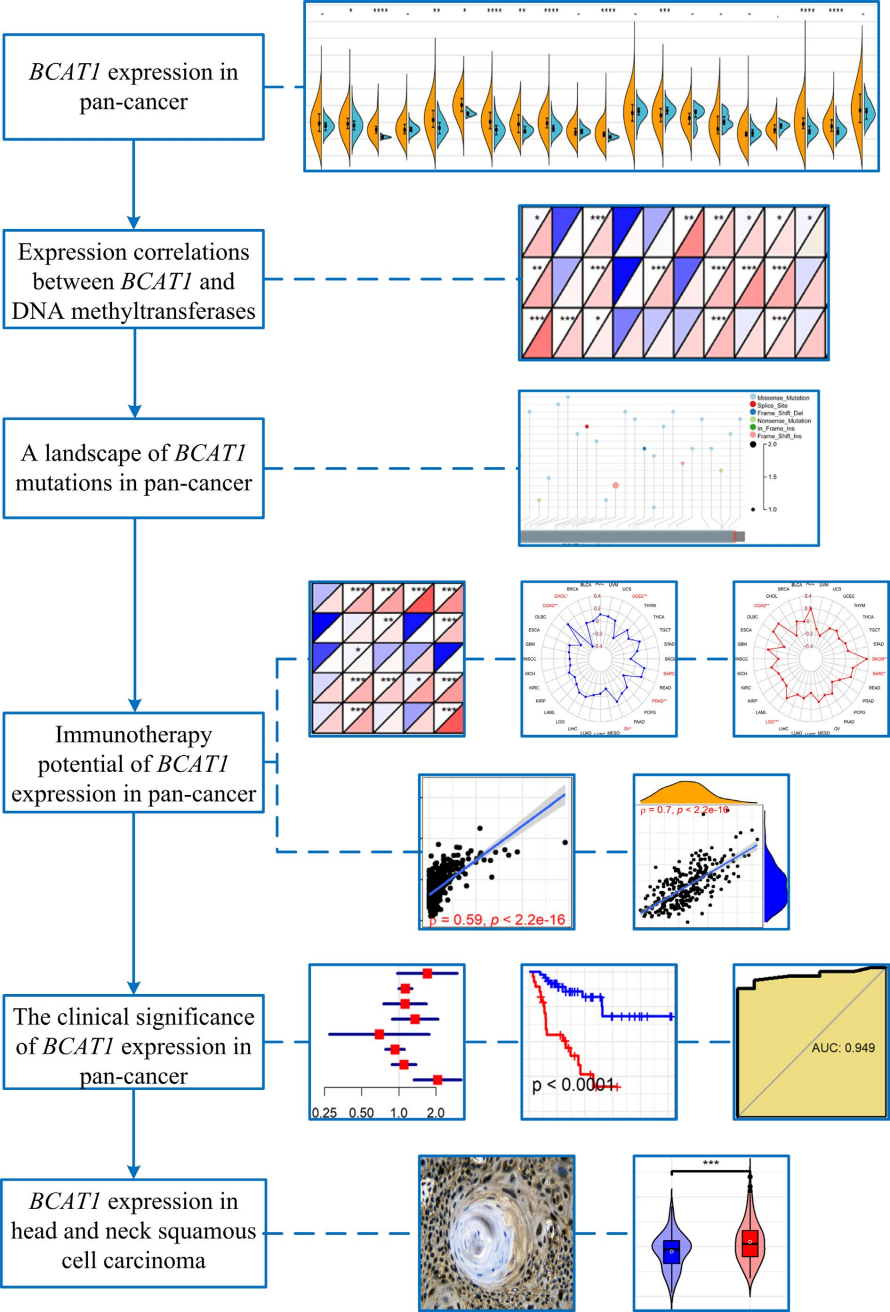
The design of this study

The result of SMD was statistically significant when the corresponding 95% confidence interval (CI) did not contain 0. Except for SMD, *p* < 0.05 indicated that the difference was statistically significant. The design of this study is shown in Figure 1. In the study, all figures containing statistical tests were generated via a series of software packages^22^, ^23^, ^24^ in R (v4.1.0).

## 3 RESULTS

### 3.1 Upregulated *BCAT1* expression in pan‐cancer

**FIGURE 2:**
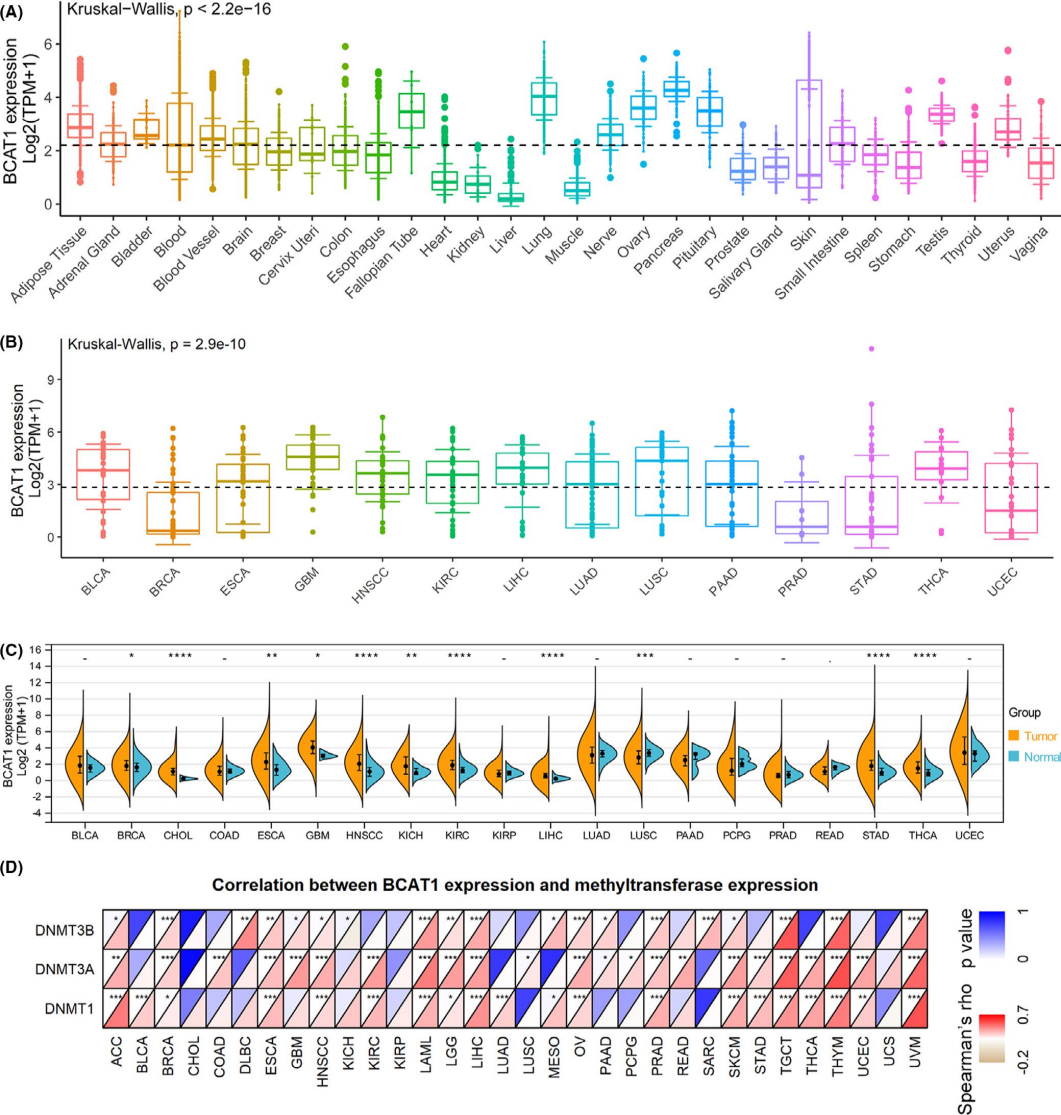
*BCAT1* expression in pan‐cancer and its correlations with DNA methyltransferases. *BCAT1* expression in normal tissues (panel A), cancer cell lines (panel B), and pan‐cancer (panel C). Panel D: Correlations between *BCAT1* expression with DNA methylation. For panel C: *p < 0.05, **p < 0.01, ***p < 0.001; *p* value was based on the Wilcoxon test

The expression of *BCAT1* was diverse in various normal tissues (Figure 2A), and the same phenomenon was observed in the cell lines of 14 cancers (Figure 2B). Furthermore, compared to corresponding normal tissues, *BCAT1* was differentially expressed in multiple cancer tissues based on TCGA data. Overexpression of *BCAT1* was detected in 10 cancers, containing breast invasive carcinoma (BRCA), cholangiocarcinoma (CHOL), esophageal carcinoma (ESCA), glioblastoma multiforme, HNSCC, kidney chromophobe, kidney renal clear cell carcinoma (KIRC), liver hepatocellular carcinoma (LIHC), stomach adenocarcinoma (STAD), and thyroid carcinoma (THCA; Figure 2C). In contrast, low *BCAT1* expression was observed in lung squamous cell carcinoma (LUSC; Figure 2C).

### 3.2 Expression correlation between *BCAT1* and DNA methyltransferases, and the landscape of *BCAT1* mutations

**FIGURE 3:**
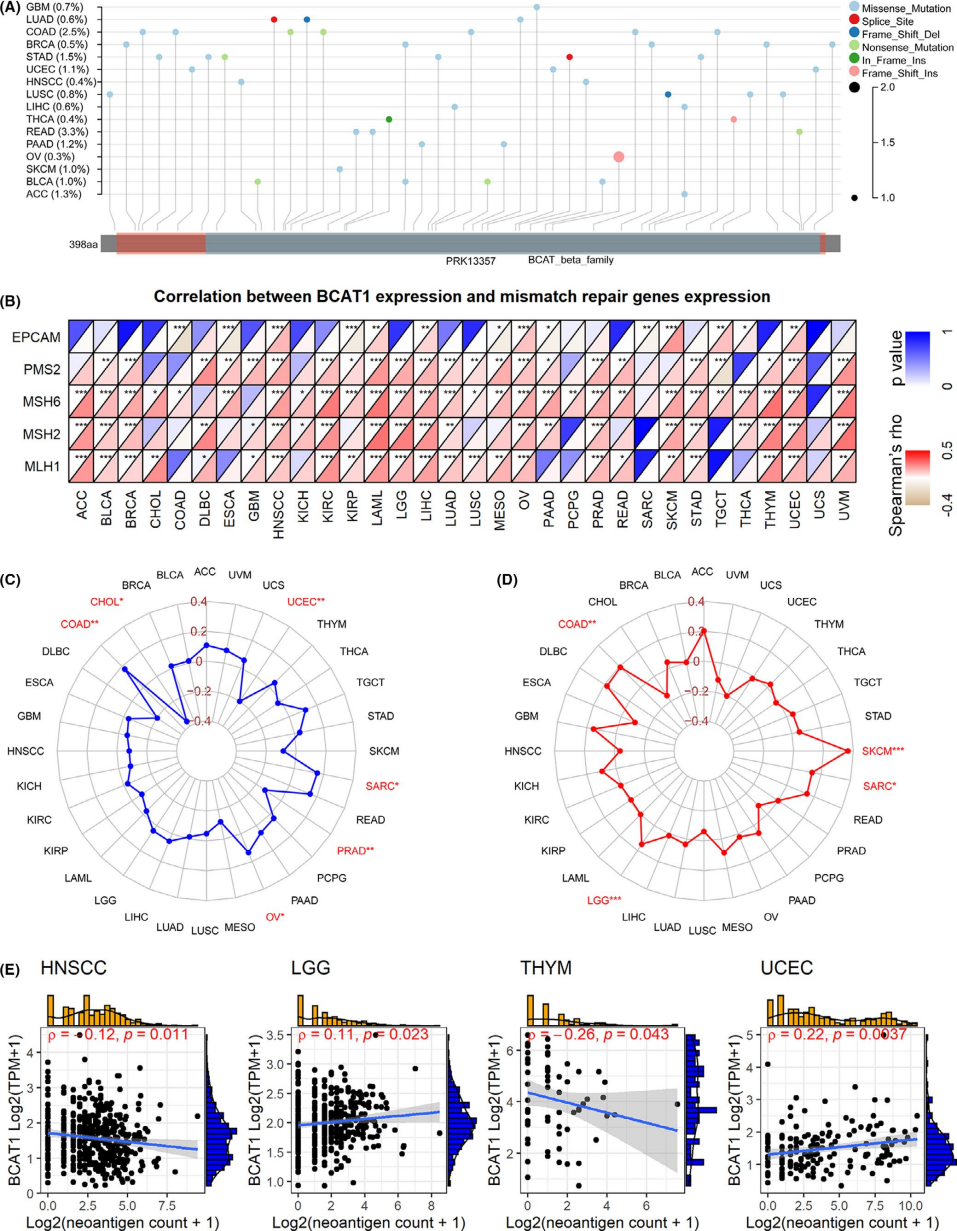
A landscape of *BCAT1* mutations, and exploration of immunotherapy potential of *BCAT1* expression in pan‐cancer. Spearman coefficient of *BCAT1* expression with mismatch repair genes expression (panel B), microsatellite instability (panel C), tumor mutational burden (panel D), and neoantigens count (panel E). For panel A: Del, deletion; Ins, insertion

As a DNA chemical modification, DNA methylation can change genetic performance without affecting DNA sequence.^25^, ^26^ Significant correlations of *BCAT1* expression with the three methyltransferases—*DNMT1*, *DNMT3A*, and *DNMT3B*—were detected in some cancers (Figure 2D). For *BCAT1*, the most common mutation in cancers was missense mutation, and not less than 2.5% of patients with colon adenocarcinoma (COAD) and rectum adenocarcinoma (READ) had observed mutations (Figure 3A).

### 3.3 Expression relationship of *BCAT1* with MMR and immune checkpoint genes expression

MMR is a repair mechanism of DNA mismatch. For cells, the dysfunction of MMR genes tends to cause difficulty in repairing DNA mismatch. As a result, the abnormal function of MMR genes can eventually lead to somatic mutations. *MLH1*, *MSH2*, *MSH6*, *PMS2*, and *EPCAM* are representative of MMR genes.^27^ In our study, conspicuous positive associations between *BCAT1* expression and the five MMR genes can be detected in quite a few cancers, particularly for HNSCC, acute myeloid leukemia, LIHC, ovarian serous cystadenocarcinoma (OV), and uterine corpus endometrial carcinoma (UCEC; Figure 3B).

Immune checkpoints are a class of immunosuppressive molecules. Tumor cells can inhibit the immune response by activating these molecules.^28^ Interestingly, *BCAT1* expression was associated with at least six immune checkpoint genes in 32 cancers, while all 46 immune checkpoint genes were detected as relevant to *BCAT1* expression in at least eight cancers (Appendix S4).

### 3.4 Correlation of *BCAT1* expression with MSI, TMB, neoantigens count, and immune microenvironment

The microsatellite is a short tandem repeat DNA sequence in the genome. A new microsatellite allele (compared to normal tissue) may occur when repeat units (resulting from MMR) are inserted or deleted at one or some microsatellite loci in tumor tissue, which is defined as MSI.^29^, ^30^ *BCAT1* expression was positively related to MSI in COAD, OV, and sarcoma (SARC), while it was negatively related to CHOL, prostate adenocarcinoma, and UCEC (Figure 3C).

TMB is an indicator used to quantitatively evaluate the total number of mutations in tumor cells. *BCAT1* expression was significantly and positively associated with TMB in four cancers, including COAD, brain lower grade glioma (LGG), SARC, and skin cutaneous melanoma (SKCM; Figure 3D).

Increasing mutations (e.g., single‐nucleotide mutations) represent more significant immunogenicity of the neoantigens encoded by cell mutant genes,^31^ and the latter can activate and proliferate T cells through complex mechanisms.^32^ Briefly, the presence of neoantigens on the surface of tumor cells helps the immune system recognize tumor cells. In our study, slight correlations between *BCAT1* expression and neoantigen count were detected in thymoma and UCEC (Figure 3E).

**FIGURE 4:**
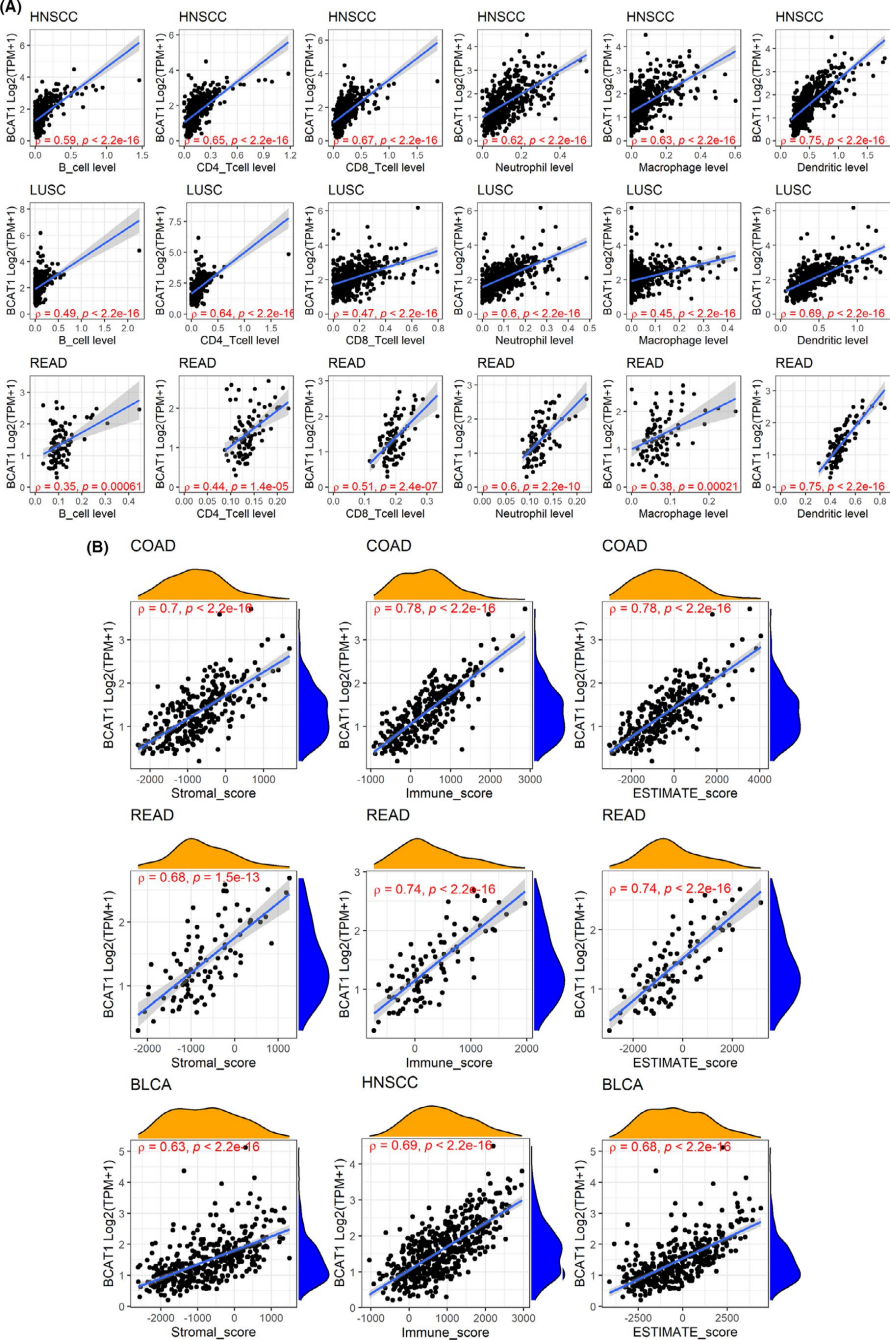
Relevance between *BCAT1* expression with infiltration levels of all the six immune cells (panel A) and immune microenvironment scores (panel B). The letter “ρ” is followed by the Spearman correlation coefficient

HNSCC, LUSC, and READ were the three cancers (HNSCC was the top one) with the most conspicuous correlations between *BCAT1* expression and infiltration levels of all six immune cells, particularly for dendritic and neutrophilic cells (Figure 4A). The most significant relationships between *BCAT1* expression and stromal scores were detected in COAD, READ, and BLCA. In three cancers––COAD, READ, and HNSCC, *BCAT1* expression showed the most conspicuous with immune scores; that for ESTIMATE scores were COAD, READ, and bladder urothelial carcinoma (BLCA; Figure 4B).

### 3.5 Relationship between *BCAT1* expression and clinical features

**FIGURE 5:**
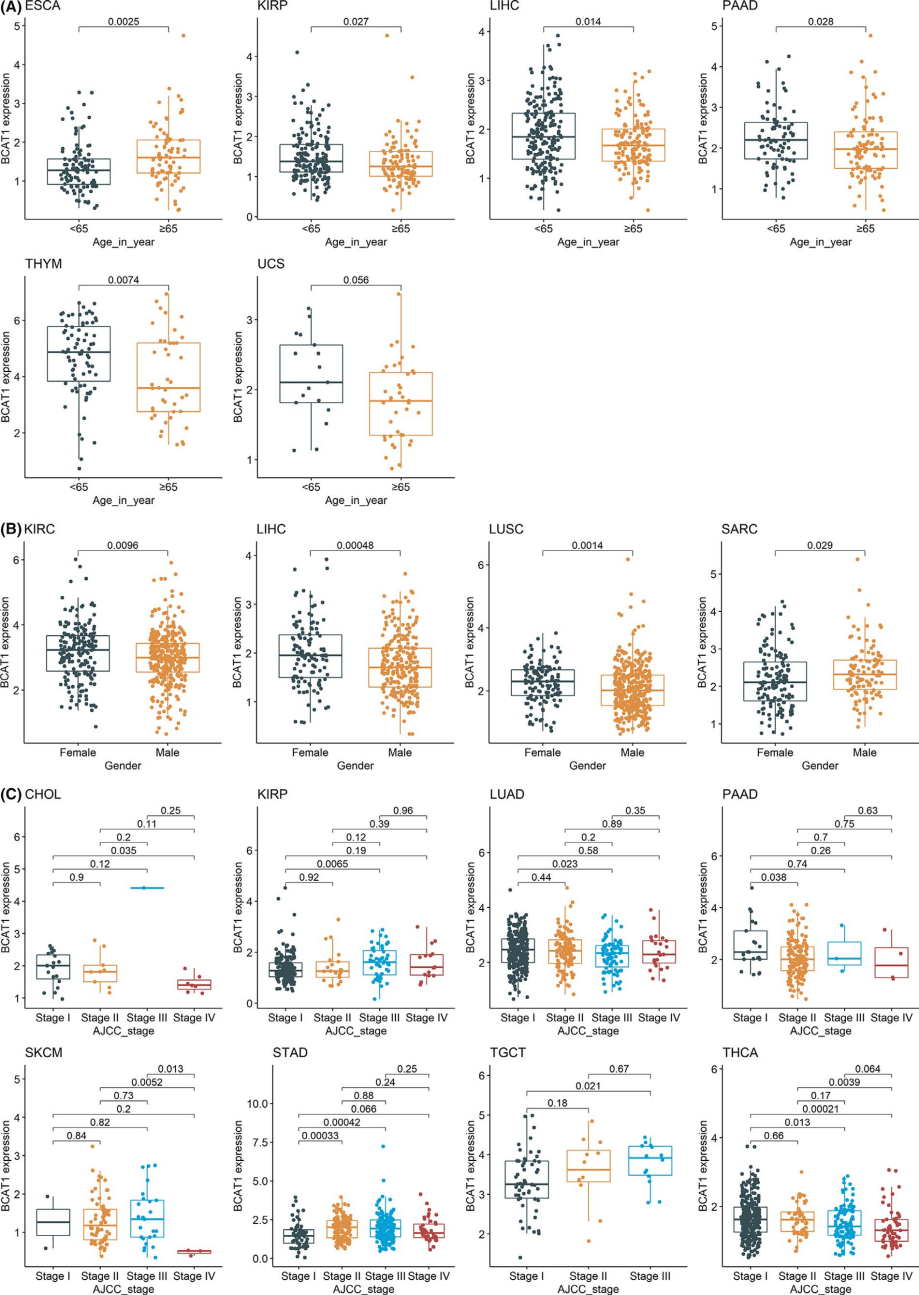
Associations of *BCAT1* expression and patients’ clinical features. The figure shows the relevance of *BCAT1* expression with age (panel A), gender (panel B), and AJCC (American Joint Committee on Cancer) stage (panel C) of cancer patients. *p* value was based on the Wilcoxon test

Tumor patients with distinct clinical features may have different prognoses, so we explored the correlation between *BCAT1* expression and clinical features. In cancers with statistically significant results (*p* < 0.05), downregulated *BCAT1* expression was detected in elderly patients (except for ESCA; Figure 5A) and males (except for SARC; Figure 5B). At the advanced stages (American Joint Committee on Cancer), *BCAT1* demonstrated diverse expression levels in various cancers—increased levels in kidney renal papillary cell carcinoma, STAD, and testicular germ cell tumors; reduced levels in CHOL, lung adenocarcinoma, pancreatic adenocarcinoma (PAAD), SKCM, and THCA (Figure 5C), suggesting that *BCAT1* may play different roles in these cancers.

### 3.6 Clinical significance of *BCAT1* expression in pan‐cancer

**FIGURE 6:**
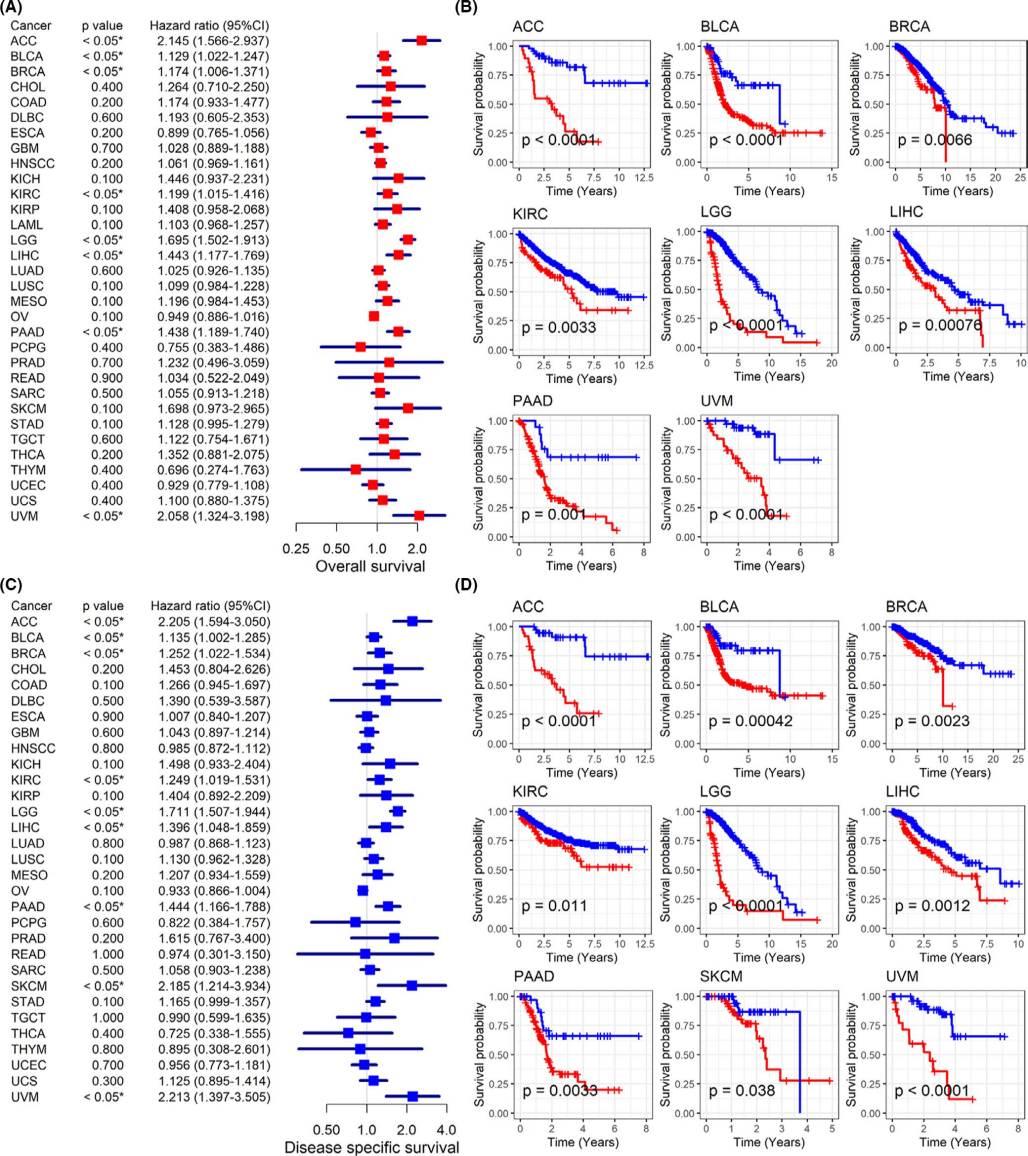
Relation of *BCAT1* expression with overall survival and disease‐specific survival of cancers patients. Panels A–B: *BCAT1* expression shows the risk factor for cancer patients (panel A) and it was related to the shorter overall survival time of cancer patients (panel B). Panels C–D: *BCAT1* expression demonstrates the risk factor for cancer patients (panel C) and it was related to shorter disease‐specific survival time of cancers patients (panel D)

**FIGURE 7:**
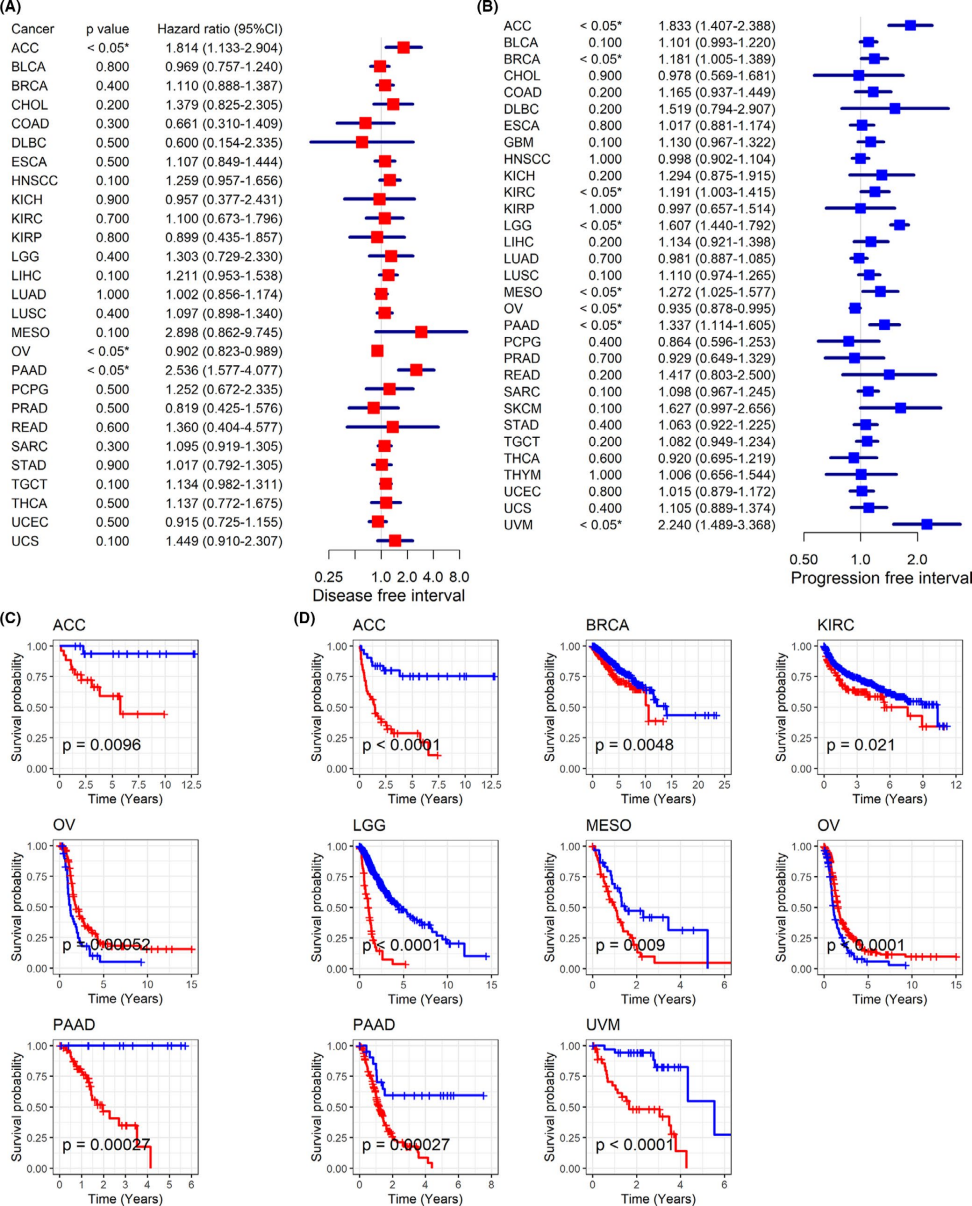
Relation of *BCAT1* expression with disease‐free survival and progression‐free survival of cancers patients. Panels A–B: *BCAT1* expression demonstrates the risk factor for most cancer patients in both disease‐free survival (panel A) and progression‐free survival (panel B). Panels C–D: *BCAT1* expression was related to shorter disease‐free survival time (panel C) and progression‐free survival time (panel D) of most cancer patients

Little was understood about the clinical value of *BCAT1* expression in multiple cancers, and thus we attempted to discuss the issue in our study. Through univariate Cox analysis, *BCAT1* expression showed the risk factor of prognostic significance in eight cancers, containing adrenocortical carcinoma (ACC), BLCA, BRCA, KIRC, LGG, LIHC, PAAD, and uveal melanoma (UVM; Figure 6A). These results were verified using the Kaplan–Meier curves, where high *BCAT1* expression represented shorter OST for patients with the eight cancers (Figure 6B). Relevance between high *BCAT1* expression and shorter DSST can be observed in ACC, BLCA, BRCA, KIRC, LGG, LIHC, PAAD, SKCM, and UVM (Figure 6C,D). Upregulated *BCAT1* expression indicated unfavorable DFIT for ACC and PAAD (Figure 7A,C). There was a similar association between *BCAT1* expression and PFIT for patients with ACC, BRCA, KIRC, LGG, mesothelioma, PAAD, and UVM (Figure 7B,D). In contrast, *BCAT1* expression tended to have a protective role for patients with OV in both DFIT and PFIT (Figure 7).

**FIGURE 8:**
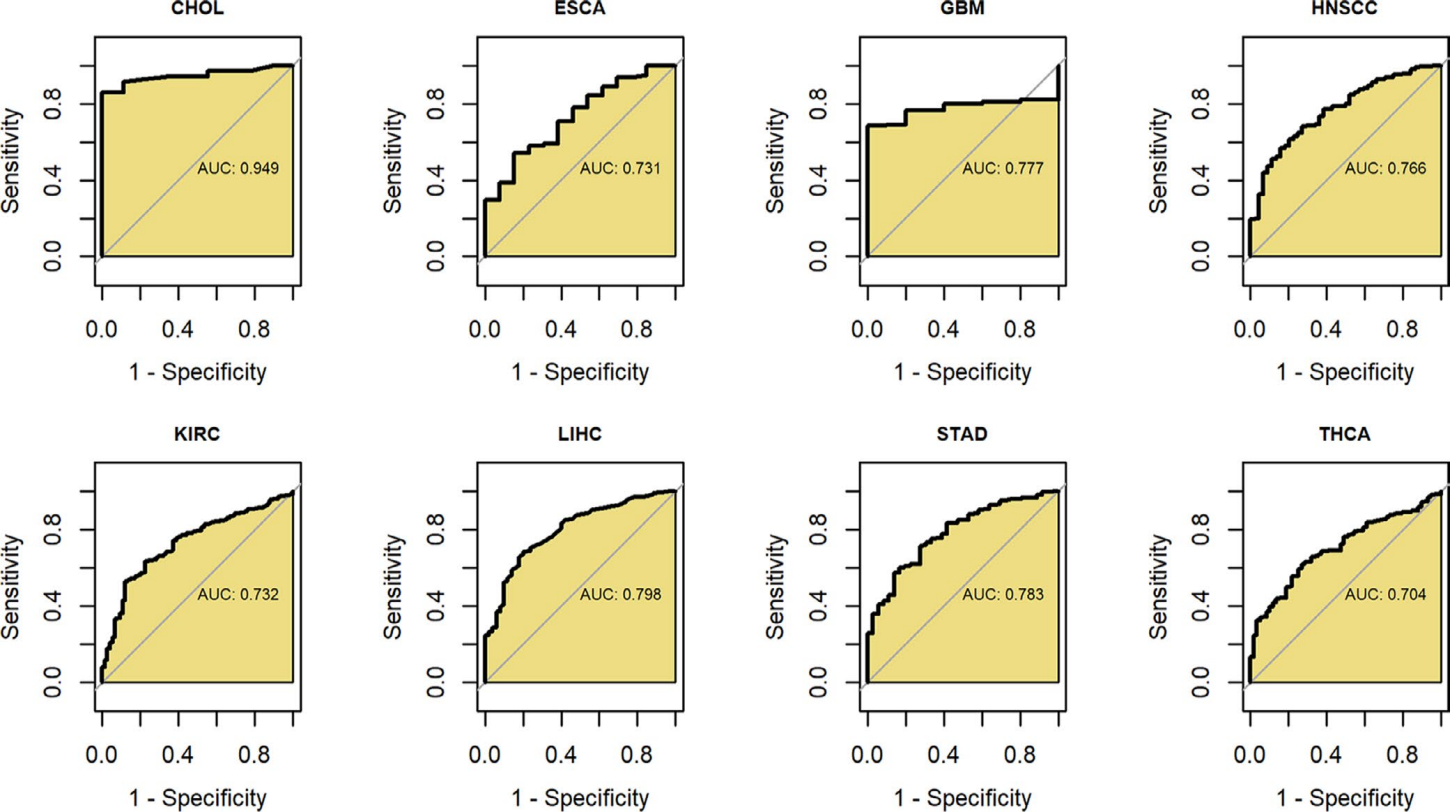
Receiver operating characteristic curves for detecting the ability of *BCAT1* expression to distinguish these cancers tissues from their normal tissues. AUC, area under the receiver operating characteristic curve

AUC values were greater than 0.7 in 8/20 cancers (Figure 8), suggesting that *BCAT1* expression demonstrated the conspicuous ability to distinguish these cancers tissues from their normal tissues. Particularly, the AUC of CHOL was up to 0.949 (Figure 8), indicating that *BCAT1* made it feasible to identify CHOL and non‐CHOL.

### 3.7 *BCAT1* expression in HNSCC

**FIGURE 9:**
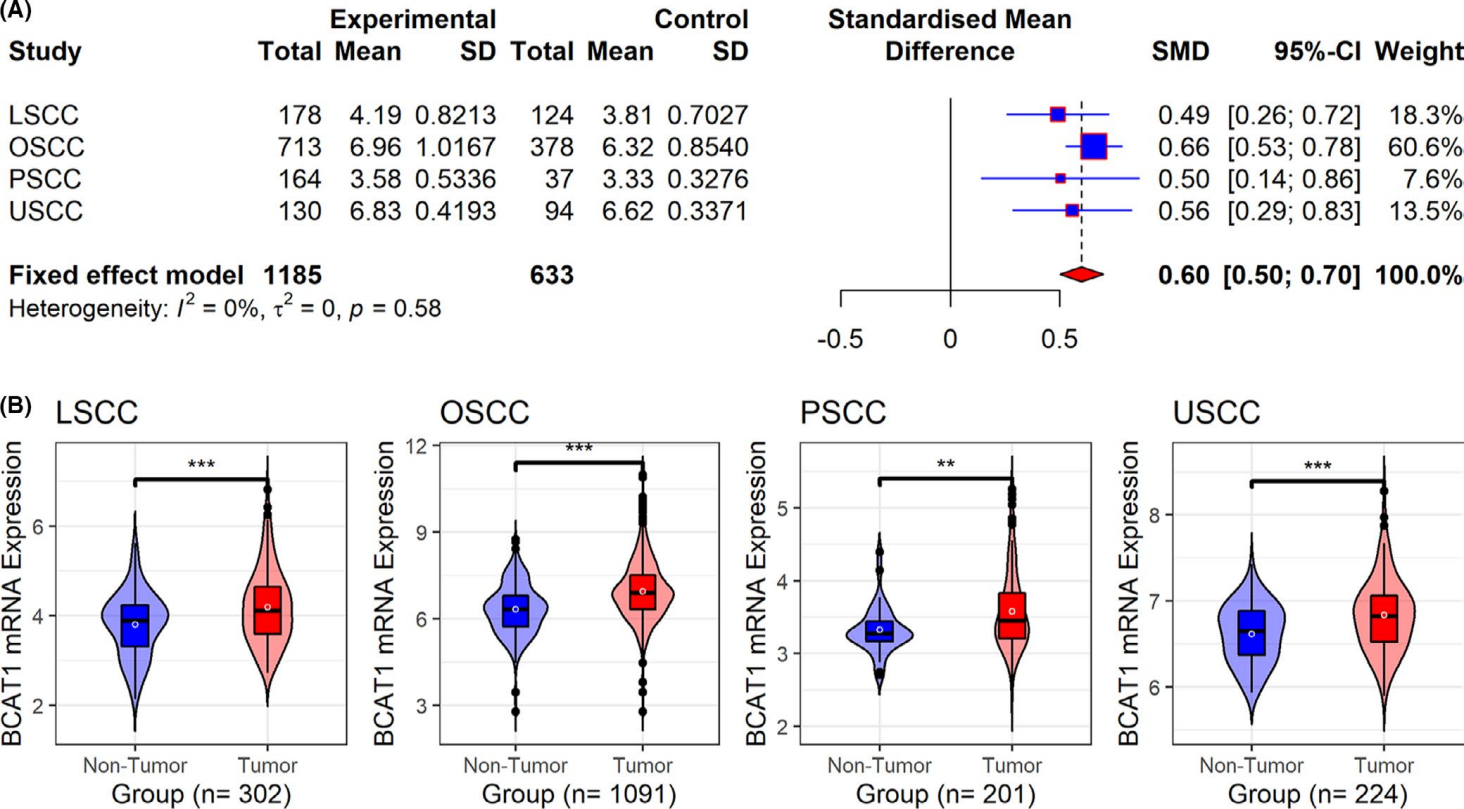
Violin plots (panel A) and the forest plot (panel B) of *BCAT1* expression in head and neck squamous cell carcinoma. LSCC: laryngeal squamous cell carcinoma; OSCC: oral squamous cell carcinoma; PSCC: pharyngeal squamous cell carcinoma; USCC: unspecific site head and neck squamous cell carcinoma. ^**^ *p* value of the Wilcoxon test <0.01, ^***^ *p* value of the Wilcoxon test <0.001

**FIGURE 10:**
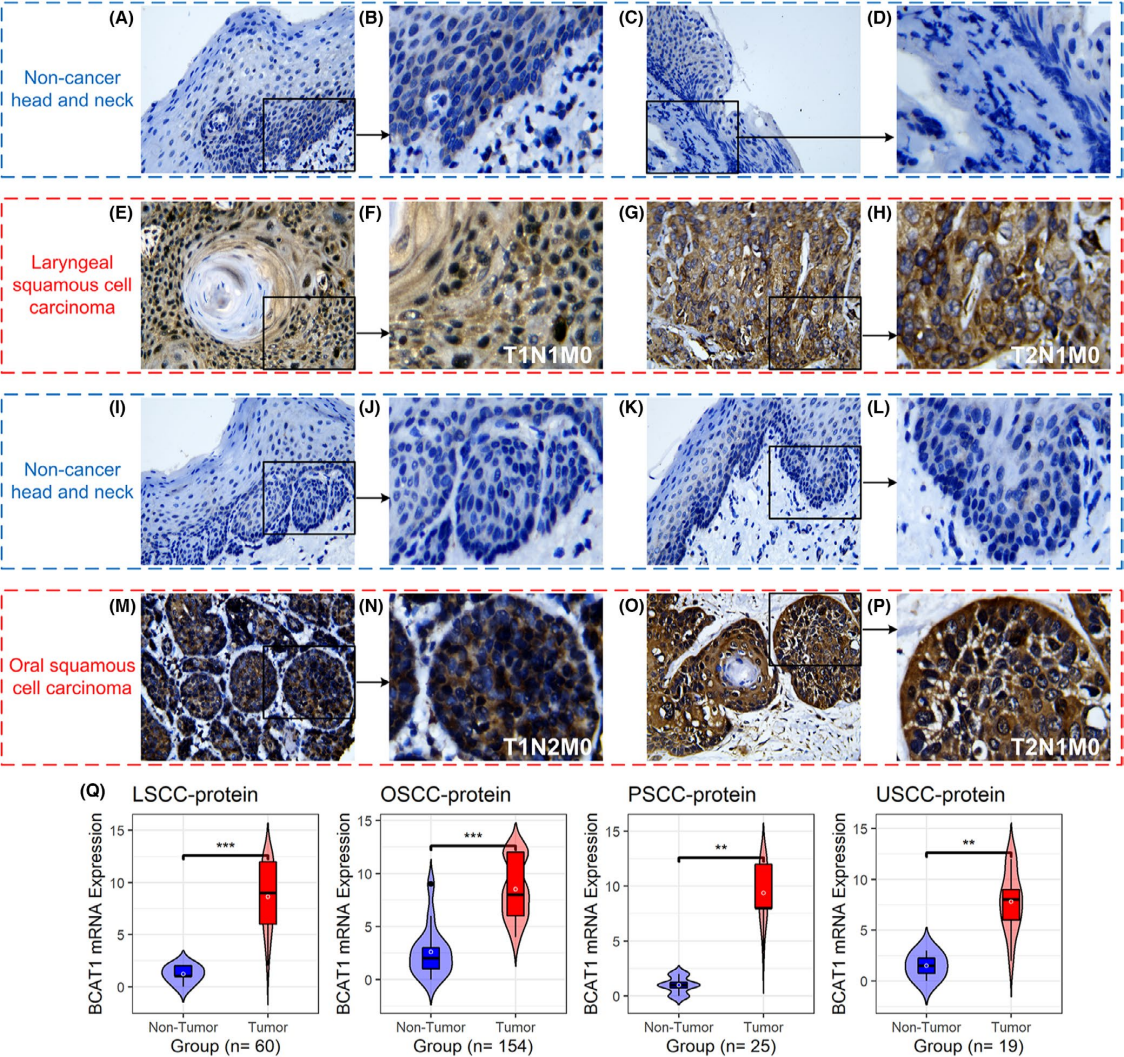
BCAT1 protein levels in tissues by in‐house tissue microarrays. Panels A–P: The protein levels of *BCAT1* in head and neck squamous cell carcinoma tissues and their control tissues under the microscope. Panel Q: A violin plot of BCAT1 protein levels in head and neck squamous cell carcinoma tissues and their control tissues. ^**^ *p* value of the Wilcoxon test <0.01, ^***^ *p* value of the Wilcoxon test <0.001

As mentioned above, differential expression was significant in pan‐cancer. Furthermore, using multicenter data, we also constructed a comprehensive study on the expression of BCAT1 in HNSCC. Based on multicenter data, upregulated *BCAT1* mRNA expression was observed in HNSCC (SMD = 0.60, 95% CI [0.50–0.70]; Figure 9A). A similar conclusion can be drawn for LSCC, OSCC, PSCC, and USCC, with their SMDs >0 and corresponding 95% CIs >0 (Figure 9A). Results of Wilcoxon tests also supported increased *BCAT1* mRNA expression in all four subgroups of HNSCC (Figure 9B). Through in‐house tissue microarrays, conspicuously positive BCAT1 protein staining was not detected in non‐HNSCC tissues (Figure 10A–D and Figure 10I–L) but in HNSCC tissues (Figure 10E–H,M–P), demonstrating high BCAT1 protein levels in HNSCC, which was confirmed using the Wilcoxon tests (Figure 10Q).

## 4 DISCUSSION

Cancer is a serious threat to human health, and research on targeted treatment and immunotherapy is meaningful. *BCAT1* encodes a transaminase of branched chain amino acids––valine, isoleucine, and leucine. Although *BCAT1* has been identified as playing an important role in a variety of tumors, no studies on *BCAT1* in pan‐cancer have previously been reported.

To the best of our knowledge, this is the first comprehensive analysis of the expression, potential mechanisms, and clinical significance of *BCAT1* in pan‐cancer, using 16,847 samples, and provides novel clues for the treatment of cancers. Differential expression of *BCAT1* was detected in various cancers, and it was relevant to some DNA methyltransferases expression, immune checkpoint genes expression, MMR genes expression, MSI, TMB, and neoantigens count in some cancers. *BCAT1* expression showed significant prognosis and identification potential in multiple cancers. To some extent, further analyses of *BCAT1* in HNSCC support some findings in pan‐cancer: upregulated BCAT1 expression (at both mRNA and protein levels) in HNSCC was identified based on in‐house tissue microarrays and datasets from various sources.

The differential expression of *BCAT1* in cancers is common, and its elevated expression is predominant. Previously, high *BCAT1* expression was reported in multiple cancers, such as ESCA,^26^ gastric cancer,^7^ ovarian cancer,^33^ and non‐small cell lung cancer.^34^ In our study, upregulated *BCAT1* expression was identified in 10 cancers (e.g., BRCA), while decreased *BCAT1* expression was detected in LUSC. Among these cancers, to the best of our knowledge, our study is the first to shed light on the diverse expression of *BCAT1* in CHOL, kidney chromophobe, STAD, and thyroid carcinoma, suggesting the novelty of our research.

*BCAT1* expression shows different correlations with DNA methyltransferases in diverse tumors. As one DNA chemical modification, DNA methylation can change genetic performance without affecting DNA sequence, where DNA methyltransferases involve.^25^ Zeng et al.^26^ identified that *DNMT1* mediated the methylation of hsa‐miR‐124‐3p, subsequently contributing to the high expression of *BCAT1* in ESCA, suggesting the correlation between *BCAT1* differential expression and DNA methylation. In our research, *BCAT1* expression was correlated with the three DNA methyltransferase genes––*DNMT1*, *DNMT3A*, and *DNMT3B* in some cancers. However, no previous reports about the relation of *BCAT1* with *DNMT3A* and *DNMT3B* were found. Therefore, it is necessary to further explore this to understand whether the three methylation genes have a regulatory relationship with the upregulated expression of *BCAT1*.

The associations of *BCAT1* expression with its mutations were found in some cancers, and dysregulated MMR genes may take part in *BCAT1* mutations. Taking COAD as an example, it had the most *BCAT1* mutations among the 32 cancers included in our study, and interestingly, *BCAT1* expression was negatively relevant to the expression of an MMR gene––*EPCAM*. Although this requires further experimental confirmation, it may make sense with the fact that: MMR is the DNA replication repair mechanism of cells, critical to ensuring genomic integrity and stability, and the dysfunction of MMR causes the deletion of DNA replication repair, resulting in gene mutations.^35^ However, more work must be carried out, focusing particularly on the relation of *BCAT1* with its mutations and MMR; doing so may contribute to the understanding of dysregulated *BCAT1* expression in pan‐cancer.

*BCAT1* may have the potential as a therapeutic target for cancers. Immune checkpoint inhibitors help the body recover a normal antitumor immune response, which is considered to be an antitumor immunotherapy. High MSI and TMB were identified as biomarkers for immune checkpoint blockade therapy, suggesting their potential in immunotherapy.^36^, ^37^ TMB contributed to the increasing neoantigens count. With neoantigens, cancer cells tend to be recognized and cleared in the immune microenvironment, where many immune cells (particularly T cells) involve.^38^ In our study, *BCAT1* expression was found to be related to MSI, TMB, and immune checkpoint genes in several cancers (e.g., COAD and SARC). Although no conspicuous relevance was found between *BCAT1* expression with neoantigens count, its remarkable correlation with the immune microenvironment was discovered in our study. For example, *BCAT1* expression was dramatically and positively related to infiltration levels of neutrophil, dendritic cells, CD4 T cells, and CD8 T cells in some cancers (e.g., HNSCC). These cells are known to take part in innate immune response and/or adaptive immune response.^39^, ^40^, ^41^ The close positive relevance of *BCAT1* expression to several immune scores was also detected in pan‐cancer analyses. Taken together, *BCAT1* is likely to be a tumor biomarker related to tumor immune cell infiltration, and it demonstrates the potential of immune treatment for multiple cancers.

*BCAT1* expression showed conspicuous clinical significance in cancers. Previously, several studies have revealed the risk role of high expression of *BCAT1* in the prognosis of patients with glioma,^11^ prostate cancer,^12^ urothelial cancer,^13^ and HNSCC.^42^ This scenario was observed in ACC, BLCA, BRCA, KIRC, LGG, LIHC, PAAD, and UVM via analysis of relevance between *BCAT1* expression and OST. Furthermore, the high *BCAT1* expression represents shorter DSST for patients with the eight cancers (ACC, etc.) and SKCM, clearly suggesting its risk roles in these cancers. As reported by Panosyan et al.,^43^ *BCAT1* was related to the adverse outcomes of glioblastoma patients. Zheng et al.^10^ identified the relationship between *BCAT1* expression and poor prognosis for LIHC, resulting from the stimulation of epithelial–mesenchymal transition by *BCAT1*.^44^ Interestingly, to the best of our knowledge, the current study is the first to reveal the prognostic significance of *BCAT1* expression in ACC, BRCA, KIRC, PAAD, and UVM, suggesting the novelty of our research. Moreover, the relevance between high *BCAT1* expression and shorter DFIT can be observed in ACC and PAAD, while the upregulated expression of *BCAT1* was relevant to poor PFIT for patients with ACC, BRCA, KIRC, LGG, mesothelioma, PAAD, and UVM. The results revealed that *BCAT1* expression represented unfavorable progression (e.g., recurrence) in patients with these cancers. Except for HNSCC,^45^, ^46^ no similar reports could thus far be found. Thus, the conspicuous and consistent risk factor of *BCAT1* expression was identified in multiple cancers. Moreover, the study revealed the significance of *BCAT1* expression in differentiating some cancers (e.g., CHOL) from their control tissues, and such a finding has not before been reported. Collectively, *BCAT1* expression was possibly a marker for prognosis and identifying cancers.

We further analyzed BCAT1 in HNSCC. Elevated *BCAT1* expression in HNSCC has been reported by Wang et al.^45^ However, some limitations like limited samples (*n* < 380) can be seen in the study. Moreover, HNSCC is complex given its multiple sites sources; for instance, a gene may be upregulated in some subgroups but not in the other subtypes.^47^, ^48^ Through comprehensively investigating a large sample (*n* of public data = 1818, *n* of in‐house tissue microarrays = 258), our study demonstrated that BCAT1 was significantly highly expressed in HNSCC at both the mRNA and protein levels in all four subgroups of HNSCC—LSCC, OSCC, PSCC, and USCC. This suggests the trend of BCAT1 expression in HNSCC is consistent with the majority of tumors (ACC, etc.).

There are some limitations to the current research. For instance, we failed to collect enough body fluid samples to verify the expression of *BCAT1* in pan‐cancer. In addition, further in vivo and in vitro experiments should be undertaken to investigate the mechanisms of *BCAT1* in pan‐cancer. In‐house samples for multiple cancers (not only HNSCC) should be included in future studies.

For the first time, this study comprehensively demonstrates a high expression of *BCAT1* in pan‐cancer, which improves the understanding of the pathogenesis of *BCAT1* in pan‐cancer. Upregulated *BCAT1* expression represents poor prognosis of cancers patients, and it serves as a potential marker for cancer immunotherapy.

## ETHICAL APPROVAL STATEMENT

The study was approved by the Ethics Committee of the First Affiliated Hospital of Guangxi Medical University, China.

## CONFLICT OF INTEREST

No potential competing interest was reported by the authors.

## AUTHOR CONTRIBUTIONS

Guo‐Sheng Li, Gang Chen, and Lin‐Jie Yang contributed to the collection of funding. All authors contributed to conception and design, drafting, and critically revising the manuscript, and gave final approval to the version to be published.

## Supporting information

Appendix S1

Appendix S2

Appendix S3

Appendix S4

## Data Availability

The data that support the findings of pan‐cancer analyses are available in public databases with serial number for each dataset (e.g., GSE107591), and the databases containing GTEx Portal at https://gtexportal.org/home/, DepMap Portal at https://depmap.org/portal/download/, Gene Expression Omnibus at https://www.ncbi.nlm.nih.gov/gds/, the Cancer Genome Atlas at https://www.cancer.gov/about‐nci/organization/ccg/research/structural‐genomics/tcga, and Sanger Box 3.0 at http://vip.sangerbox.com/. Data on in‐house tissue microarrays and microarray matrix used during the current study are available from the corresponding author upon reasonable request.
