# Supplementary material for: BCAT1: A risk factor in multiple cancers based on a pan‐cancer analysis
Source: Cancer Med. 2022 Jan 4;11(5):1396–412. doi: 10.1002/cam4.4525 (PMC8894718; doi:10.1002/cam4.4525)
Supplement: Supplementary file 1 — Appendix S1 [file CAM4-11-1396-s002.docx]

**Appendix 1.** Abbreviation of cancers name and number of samples for each cancer.

| Cancer abbreviation | Cancer full name | *n* of cancer group | *n* of control group |
| --- | --- | --- | --- |
| ACC | Adrenocortical Carcinoma | 77 | 0 |
| BLCA | Bladder Urothelial Carcinoma | 407 | 19 |
| BRCA | Breast Invasive Carcinoma | 1092 | 113 |
| CHOL | Cholangiocarcinoma | 36 | 9 |
| COAD | Colon Adenocarcinoma | 288 | 41 |
| DLBC | Lymphoid Neoplasm Diffuse Large B-Cell Lymphoma | 47 | 0 |
| ESCA | Esophageal Carcinoma | 181 | 13 |
| GBM | Glioblastoma Multiforme | 153 | 5 |
| HNSCC | Head And Neck Squamous Cell Carcinoma | 518 | 44 |
| KICH | Kidney Chromophobe | 66 | 25 |
| KIRC | Kidney Renal Clear Cell Carcinoma | 530 | 75 |
| KIRP | Kidney Renal Papillary Cell Carcinoma | 288 | 32 |
| LAML | Acute Myeloid Leukemia | 173 | 0 |
| LGG | Brain Lower Grade Glioma | 509 | 0 |
| LIHC | Liver Hepatocellular Carcinoma | 369 | 50 |
| LUAD | Lung Adenocarcinoma | 513 | 59 |
| LUSC | Lung Squamous Cell Carcinoma | 498 | 50 |
| MESO | Mesothelioma | 87 | 0 |
| OV | Ovarian Serous Cystadenocarcinoma | 419 | 0 |
| PAAD | Pancreatic Adenocarcinoma | 178 | 4 |
| PCPG | Pheochromocytoma And Paraganglioma | 177 | 3 |
| PRAD | Prostate Adenocarcinoma | 495 | 52 |
| READ | Rectum Adenocarcinoma | 92 | 10 |
| SARC | Sarcoma | 258 | 2 |
| SKCM | Skin Cutaneous Melanoma | 102 | 1 |
| STAD | Stomach Adenocarcinoma | 414 | 36 |
| TGCT | Testicular Germ Cell Tumors | 148 | 0 |
| THCA | Thyroid Carcinoma | 504 | 59 |
| THYM | Thymoma | 119 | 2 |
| UCEC | Uterine Corpus Endometrial Carcinoma | 180 | 23 |
| UCS | Uterine Carcinosarcoma | 57 | 0 |
| UVM | Uveal Melanoma | 79 | 0 |
