# Supplementary material for: BCAT1: A risk factor in multiple cancers based on a pan‐cancer analysis
Source: Cancer Med. 2022 Jan 4;11(5):1396–412. doi: 10.1002/cam4.4525 (PMC8894718; doi:10.1002/cam4.4525)
Supplement: Supplementary file 2 — Appendix S2 [file CAM4-11-1396-s004.docx]

**Appendix 2.** The serial number and the sample size for included data sets related to head and neck squamous carcinoma (HNSCC).

| Subgroup | Serial number | Sample size | | Total sample size |
| --- | --- | --- | --- | --- |
|  |  | Non-HNSCC | HNSCC |  |
| LSCC^a^ | GSE107591-LSCC | 4 | 4 | 8 |
|  | GSE10774-LSCC | 0 | 2 | 2 |
|  | GSE117005 | 5 | 5 | 10 |
|  | GSE123986 | 0 | 35 | 35 |
|  | GSE127165 | 57 | 57 | 114 |
|  | GSE137308 | 3 | 3 | 6 |
|  | GSE143224 | 11 | 14 | 25 |
|  | GSE29330 | 5 | 3 | 8 |
|  | GSE51985 | 10 | 10 | 20 |
|  | GSE58911-LSCC | 7 | 7 | 14 |
|  | GSE59102 | 13 | 29 | 42 |
|  | GSE84957 | 9 | 9 | 18 |
| OSCC^b^ | GSE10121 | 6 | 35 | 41 |
|  | GSE107591-OSCC | 16 | 17 | 33 |
|  | GSE10774-OSCC | 0 | 4 | 4 |
|  | GSE135975 | 3 | 8 | 11 |
|  | GSE13601 | 27 | 31 | 58 |
|  | GSE138206 | 12 | 6 | 18 |
|  | GSE140707 | 3 | 3 | 6 |
|  | GSE146483 | 3 | 8 | 11 |
|  | GSE150469 | 2 | 5 | 7 |
|  | GSE19089 | 3 | 3 | 6 |
|  | GSE23558 | 5 | 27 | 32 |
|  | GSE25099 | 22 | 57 | 79 |
|  | GSE30784 | 62 | 167 | 229 |
|  | GSE31056 | 73 | 23 | 96 |
|  | GSE34106 | 15 | 28 | 43 |
|  | GSE3524 | 4 | 16 | 20 |
|  | GSE36090 | 4 | 20 | 24 |
|  | GSE37991 | 40 | 40 | 80 |
|  | GSE51010 | 8 | 48 | 56 |
|  | GSE56532 | 6 | 10 | 16 |
|  | GSE58911-OSCC | 4 | 4 | 8 |
|  | GSE74530 | 6 | 6 | 12 |
|  | GSE75538 | 14 | 14 | 28 |
|  | GSE75539 | 8 | 7 | 15 |
|  | GSE78060 | 4 | 26 | 30 |
|  | GSE85195 | 16 | 34 | 50 |
|  | GSE89146 | 0 | 40 | 40 |
|  | GSE9844 | 12 | 26 | 38 |
| PSCC^c^ | GSE107591-PSCC | 3 | 3 | 6 |
|  | GSE10774-PSCC | 0 | 4 | 4 |
|  | GSE13397 | 8 | 8 | 16 |
|  | GSE13398 | 8 | 8 | 16 |
|  | GSE55546 | 4 | 12 | 16 |
|  | GSE55549 | 4 | 8 | 12 |
|  | GSE55550 | 8 | 119 | 127 |
|  | GSE58911-PSCC | 2 | 2 | 4 |
| USCC^d^ | GSE33205 | 25 | 44 | 69 |
|  | GSE39400 | 11 | 28 | 39 |
|  | GSE58911-USCC | 2 | 2 | 4 |
|  | GSE6631 | 22 | 22 | 44 |
|  | GSE73330 | 12 | 12 | 24 |
|  | GSE83519 | 22 | 22 | 44 |

Notes: ^a^: laryngeal squamous cell carcinoma; ^b^: oral squamous cell carcinoma; ^c^: pharyngeal squamous cell carcinoma; ^d^: unspecific-site squamous cell carcinoma.
