# Supplementary material for: BCAT1: A risk factor in multiple cancers based on a pan‐cancer analysis
Source: Cancer Med. 2022 Jan 4;11(5):1396–412. doi: 10.1002/cam4.4525 (PMC8894718; doi:10.1002/cam4.4525)
Supplement: Supplementary file 3 — Appendix S3 [file CAM4-11-1396-s001.docx]

**Appendix 3.** Basic information of immunohistochemistry samples of each subgroup of head and neck squamous cell carcinoma.

| Clinical parameter | | Number of samples (Percentage) | | | |
| --- | --- | --- | --- | --- | --- |
|  |  | LSCC^a^ | OSCC^b^ | PSCC^c^ | USCC^d^ |
| Age (years) | <65 | 40 (72.7%) | 101 (72.7%) | 17 (80.9%) | 11 (73.3%) |
|  | ≥65 | 10 (18.2%) | 32 (23%) | 3 (14.3%) | 4 (26.7%) |
|  | NA^e^ | 5 (9.1%) | 6 (4.3%) | 1 (4.8%) | 0 (0%) |
| Gender | Female | 1 (1.8%) | 32 (23%) | 2 (9.5%) | 3 (20%) |
|  | Male | 49 (89.1%) | 101 (72.7%) | 18 (85.7%) | 12 (80%) |
|  | NA | 5 (9.1%) | 6 (4.3%) | 1 (4.8%) | 0 (0%) |
| Tumor stage | T1-2 | 30 (54.5%) | 101 (72.7%) | 9 (42.9%) | 11 (73.3%) |
|  | T3-4 | 20 (36.4%) | 32 (23%) | 11 (52.4%) | 4 (26.7%) |
|  | NA | 5 (9.1%) | 6 (4.3%) | 1 (4.8%) | 0 (0%) |
| Nodes stage | N0 | 23 (41.8%) | 78 (56.1%) | 9 (42.9%) | 12 (80%) |
|  | N1-2 | 27 (49.1%) | 55 (39.6%) | 11 (52.4%) | 3 (20%) |
|  | NA | 5 (9.1%) | 6 (4.3%) | 1 (4.8%) | 0 (0%) |
| Metastasis stage | M0 | 50 (90.9%) | 132 (95%) | 20 (95.2%) | 15 (100%) |
|  | M1 | 0 (0%) | 1 (0.7%) | 0 (0%) | 0 (0%) |
|  | NA | 5 (9.1%) | 6 (4.3%) | 1 (4.8%) | 0 (0%) |
| Clinical stage | I-II | 16 (29.1%) | 60 (43.2%) | 4 (19%) | 8 (53.3%) |
|  | III-IV | 34 (61.8%) | 79 (56.8%) | 16 (76.2%) | 7 (46.7%) |
|  | NA | 5 (9.1%) | 0 (0%) | 1 (4.8%) | 0 (0%) |

Notes: ^a^: laryngeal squamous cell carcinoma; ^b^: oral squamous cell carcinoma; ^c^: pharyngeal squamous cell carcinoma; ^d^: unspecific site squamous cell carcinoma; ^e^: not available.
